# A Recombinant Avian Infectious Bronchitis Virus Expressing a Heterologous Spike Gene Belonging to the 4/91 Serotype

**DOI:** 10.1371/journal.pone.0024352

**Published:** 2011-08-30

**Authors:** Maria Armesto, Sharon Evans, David Cavanagh, Abu-Bakr Abu-Median, Sarah Keep, Paul Britton

**Affiliations:** Avian Viral Diseases, Institute for Animal Health, Compton Laboratory, Compton, Newbury, Berkshire, United Kingdom; Kantonal Hospital St. Gallen, Switzerland

## Abstract

We have shown previously that replacement of the spike (S) gene of the apathogenic IBV strain Beau-R with that from the pathogenic strain of the same serotype, M41, resulted in an apathogenic virus, BeauR-M41(S), that conferred protection against challenge with M41 [1]. We have constructed a recombinant IBV, BeauR-4/91(S), with the genetic backbone of Beau-R but expressing the spike protein of the pathogenic IBV strain 4/91(UK), which belongs to a different serogroup as Beaudette or M41. Similar to our previous findings with BeauR-M41(S), clinical signs observations showed that the S gene of the pathogenic 4/91 virus did not confer pathogenicity to the rIBV BeauR-4/91(S). Furthermore, protection studies showed there was homologous protection; BeauR-4/91(S) conferred protection against challenge with wild type 4/91 virus as shown by the absence of clinical signs, IBV RNA assessed by qRT-PCR and the fact that no virus was isolated from tracheas removed from birds primarily infected with BeauR-4/91(S) and challenged with IBV 4/91(UK). A degree of heterologous protection against M41 challenge was observed, albeit at a lower level.

Our results confirm and extend our previous findings and conclusions that swapping of the ectodomain of the S protein is a precise and effective way of generating genetically defined candidate IBV vaccines.

## Introduction

Avian infectious bronchitis virus (IBV) is a *gammacoronavirus*, subfamily *Coronavirinae,* family *Coronaviridae*, Order *Nidovirales* [2] and is the aetiological agent of the acute highly contagious poultry disease infectious bronchitis (IB) [3]–[6]. IBV is a highly infectious pathogen of domestic fowl that replicates primarily in epithelial cells of the respiratory tract [7], [8] causing IB characterised by nasal discharge, snicking, tracheal ciliostasis and rales in chickens [1]. Although IBV is primarily associated with respiratory tract infections, it is also responsible for major economic losses to poultry industries worldwide as a result of poor weight gain and decreased egg production [9]. In addition, some isolates have been found to be associated with renal disease and can be highly nephropathogenic [10]–[12].

Coronaviruses are enveloped viruses that replicate in the cell cytoplasm and contain an unsegmented, single-stranded, positive-sense RNA genome of 28 to 32 kb [13]–[15]. IBV, like all coronaviruses, contains the four structural proteins; spike glycoprotein (S), small membrane protein (E), integral membrane protein (M) and nucleocapsid protein (N), which interacts with the genomic RNA. The coronavirus S glycoprotein is a type I glycoprotein that oligomerises in the endoplasmic reticulum [16] to form trimers [17], which constitute the coronavirus virion spikes observable by electron microscopy. The S protein is assembled into virion membranes, through non-covalent interactions with the M protein [18], and is responsible for binding to the target cell receptor and fusion of the viral and cellular membranes, fulfilling a major role in the infection of susceptible cells [19].

All coronavirus S glycoproteins consist of four domains; a signal sequence that is cleaved during synthesis; the ectodomain, which is present on the outside of the virion particle; the transmembrane region responsible for anchoring the S protein into the lipid bilayer of the virion particle; and the cytoplasmic tail. The IBV S glycoprotein (1162 amino acids) is cleaved into two subunits, S1 (535 amino acids, 90-kDa) comprising the N-terminal subunit of the S protein and S2 (627 amino acids, 84-kDa) comprising the C-terminal subunit of the S protein. The S2 subunit associates non-covalently with the S1 subunit and contains the transmembrane and C-terminal cytoplasmic tail domains. The S1 subunit contains the receptor-binding activity of the S protein [20], [21]. The ectodomain region of the S2 subunit contains a fusion peptide-like region [22] and two heptad repeat regions involved in oligomerisation of the S protein [23] and is required for entry into susceptible cells [24]–[26].

We have previously shown, using our IBV reverse genetics system [27]–[29], that replacement of the ectodomain of the IBV Beaudette S glycoprotein with the corresponding region from the pathogenic IBV M41-CK did not confer virulence to Beau-R but did result in a rIBV, BeauR-M41(S), which had the tissue tropism associated with M41-CK [30]. Chickens that were vaccinated with BeauR-M41(S) were found to be protected against clinical disease following challenge with IBV M41-CK, whereas chickens vaccinated with Beau-R were not protected against challenge with M41-CK [1]. These results indicated that BeauR-M41(S) was able to induce a protective response against homologous challenge with M41-CK, whereas Beau-R was unable to induce a protective response even though both viruses belong to the same, Massachusetts, serogroup. Beau-R is a molecular clone of the Beaudette isolate, an IBV that was attenuated after several hundred passages in embryonated hen's eggs [31], which not only resulted in loss of virulence, but has also been implicated in loss of immunogenicity. Our work has shown that infectious virus was not recovered from tracheal cells following infection of chickens with Beau-R and BeauR-M41(S) or for the H120 [32] vaccine strain of IBV [1]. The genome of BeauR-M41(S) is isogenic with Beau-R and the fact that it is able to protect against homologous challenge indicates that Beaudette or Beaudette-based viruses are able to replicate in chickens raising the possibility that the inability of Beaudette to protect against a virus from the same serotype is not due to the fact the virus cannot replicate in the chicken, but due to an immunogenic mis-match of the Beaudette S glycoprotein with other Massachusetts serotype viruses. Replication of IBV and IBV vaccines in the chicken Harderian gland has been implicated in the induction of immunity against IBV [32]–[35] indicating that IBV vaccines are able to replicate in this tissue. It is possible that some other protein derived from Beaudette in combination with the M41 S glycoprotein may overcome the block in protection associated with Beau-R. In this paper we describe the use of our IBV reverse genetics system to further investigate the role of the IBV S glycoprotein in protection. A rIBV was generated in which the ectodomain region of Beaudette S glycoprotein gene was replaced with the corresponding sequence from IBV 4/91(UK); a pathogenic strain of IBV that belongs to a different serogroup as Beaudette and M41, that has been an important problem to the poultry industry since the 1990s [6].

## Materials and Methods

### Ethics Statement

All animal experimental protocols were carried out in strict accordance with the UK Home Office guidelines and under licence granted for experiments involving regulated procedures on animals protected under the UK Animals (Scientific Procedures) Act 1986. The experiments were performed in the IAH Home Office licensed (PCD30/4301) experimental animal house facilities and were approved by the IAH ethical review committee under the terms of reference HO-ERP-01-1, using chickens obtained from the IAH Poultry Production Unit.

### Cells and viruses

The pathogenic IBV strain 4/91(UK) used in this study was a gift from Intervet UK Ltd and was grown in 10-day-old specific pathogen free (SPF) Rhode Island Red (RIR) embryonated hen's eggs obtained from the Institutes' poultry production unit; primary chick kidney (CK) cells are refractory for growth of IBV 4/91(UK). M41-CK was derived from the pathogenic M41 strain of IBV following adaptation in CK cells [36], [37]. Vaccinia viruses (VV) were routinely grown and titrated on Vero cells as described previously, whilst large stocks for DNA isolation were prepared from infected BHK-21 cells [28], [29]. Tracheal organ cultures (TOCs) were prepared from 19-day-old SPF RIR chicken embryos [36], [38]. Titrations of virus infectivity were performed in TOCs and the titres expressed as the 50% (median) ciliostatic doses (CD_50_) as described previously [1].

### Construction of chimaeric spike gene

**Figure 1:**
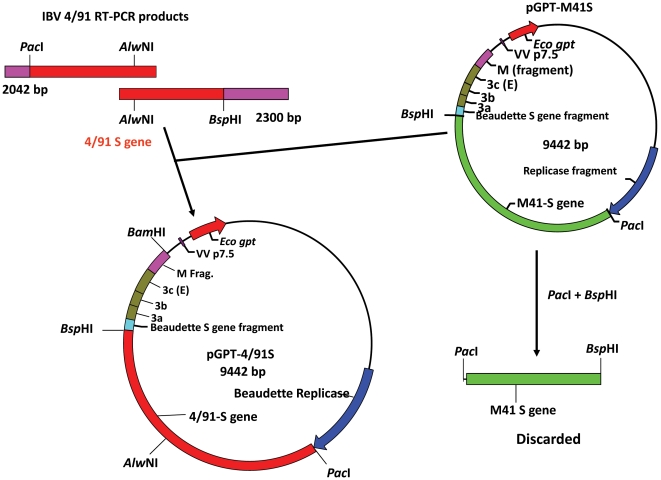
Schematic diagram for the construction of the chimaeric 4/91 S gene. Two DNA fragments of 2042 and 2300-nucleotides were generated from the IBV 4/91(UK) S gene sequence that overlapped at an internal *Alw*NI site. The 2042-nucleotide cDNA, corresponding to the 5′ half of the 4/91 S gene was digested with *Pac*I and *Alw*NI and ligated to the *Alw*NI-*Bsp*HI fragment generated from the 2300-nucleotide cDNA corresponding to the 3′ half of the 4/91 S gene. The 4/91-derived 3389-nucleotide *Pac*I-*Bsp*HI cDNA was then used to replace the corresponding M41-CK S gene sequence in pGPT-M41S generating a new chimaeric S gene sequence with the ectodomain derived from 4/91(UK) and the cytoplasmic domain from Beau-R in pGPT-4/91S. Plasmid pGPT-4/91S was used to insert the 4/91 chimaeric S gene into the Beau-R genome by homologous recombination utilising the Beau-R replicase and gene 3 sequences, 5′ and 3′ to the S gene sequence, respectively, using rVV VV-BeauR-ΔS by TDS.

The sequence corresponding to the chimaeric S gene, consisting of 3327 nucleotides from the ectodomain and transmembrane domain from IBV 4/91(UK) S gene sequence (GenBank accession number JN192154) and 136 nucleotides corresponding to the cytoplasmic domain from Beau-R (GenBank accession number AJ311317), was produced as described in Fig. 1. The 4/91-derived S gene sequence was amplified by RT-PCR using RNA extracted from the allantoic fluid of embryonated eggs infected with IBV 4/91(UK) and 4/91-derived primers containing *Pac*I (5′) and *Bsp*HI (3′) restriction sites. The chimaeric S gene was generated using the *Pac*I and *Bsp*HI sites present at the same respective positions in both virus-derived sequences (Fig. 1). The *Pac*I site at nucleotide 20337 is 30 nucleotides proximal of the S gene initiation codon and 21 nucleotides distal of the S gene transcriptional regulatory sequence, the *Bsp*HI site at nucleotide 23714 is at the end of the transmembrane domain. In brief, the 4/91-derived 3389-nucleotide *Pac*I-*Bsp*HI cDNA fragment was used to replace the corresponding M41 S sequence in pGPT-M41S [28] generating pGPT-4/91S. The pGPT-M41S vector was digested with *Pac*I and *Bsp*HI to remove the M41 sequence and used for insertion of the *Pac*I-*Bsp*HI 4/91-derived cDNA (Fig. 1). The resulting plasmid, pGPT-4/91S consisted of 1245 nucleotides of the 3′-end of the replicase gene of Beau-R, followed by the 4/91-Beaudette chimaeric S gene and 944 nucleotides of gene 3 and M gene of Beau-R (Fig. 1). RT-PCR products were sequenced using a variety of oligonucleotides covering the S gene sequence and adjoining regions of the Beaudette sequence and used for the assembly of 4/91-derived S gene sequence using Gap4 of the Staden Sequence Software Programs [39].

### Generation of recombinant vaccinia viruses containing modified IBV cDNAs

The 4/91 S sequence within pGPT-4/91S was introduced into the IBV genome within recombinant vaccinia virus (rVV) VV-BeauR-ΔS, which contains the complete Beau-R genome, minus the S sequence [28]. This was achieved by homologous recombination using the transient dominant selection system (TDS) [28]. In brief, Vero cells (∼50% confluent) were infected with VV-BeauR-ΔS at a MOI of 0.2 and transfected 2 h later with 5 µg of pGPT-4/91(S) in the presence of lipofectin (Invitrogen). Resultant phenotypically guanine xanthine phosphoribosyltransferase positive (GPT^+^) rVVs were then selected by three rounds of plaque purification using Vero cells in the presence of 25 µg/ml mycophenolic acid (MPA), 250 µg/ml xanthine and 15 µg/ml hypoxanthine. Next, randomly selected MPA-resistant GPT^+^ rVVs were grown and plaque purified three times in Vero cells in the absence of selection medium. This process resulted in a second recombination event causing the loss of the GPT gene from the rVVs; leading to either generation of rVVs containing IBV cDNA corresponding to the original sequence (BeauR-ΔS) or to a full-length BeauR-4/91(S) cDNA. PCR amplification of the DNA from these rVVs was used to (a) confirm the absence of the GPT gene, using GPT-specific primers; and (b) to determine the presence of the chimaeric S gene sequence, using 4/91-specific S gene primers. Recombinant VVs, that were GPT negative and generated PCR products of the expected size from a 4/91 S gene sequence, were further screened by sequence analysis of different regions of the S1 and S2 subunits of the chimaeric S gene to confirm that the required S gene had been introduced into the IBV genomic cDNA. A rVV, rVV-BeauR-4/91(S), which contained a full-length IBV cDNA consisting of the genomic background of Beau-R but with the ectodomain sequence of the 4/91(UK) S gene sequence, was chosen and used for further work.

### Recovery of an infectious rIBV expressing a chimaeric S protein

DNA from rVV-BeauR-4/91(S) was purified and initially used to rescue rIBVs in CK cells as previously described [28]. Cell lysate (0.1 ml) from the infected and transfected CK cells (P_0_) was used to infect 10-day-old SPF embryos. The infected embryos were incubated at 37°C for 48 h, after which they were placed at 4°C overnight. Allantoic fluid was collected (EP_1_) and was passed a further five times in 10-day-old SPF embryos and the resultant rIBV, BeauR-4/91(S), was used in subsequent experiments. RNA was extracted from the allantoic fluid of infected eggs using the RNeasy® method (Qiagen) for the amplification of part of the S gene by RT-PCR (Ready-To-Go^TM^ RT-PCR beads) to confirm the identity of the rIBV by sequence analysis. A stock of BeauR-4/91(S) was produced in 10-day-old SPF embryonated eggs, final titre 2×10^5.6^ CD_50_ per ml, which was used for subsequent *in vivo* experiments.

### *In vivo* analysis of the rIBVs

Virus stocks for the *in vivo* experiments were prepared from 10-day-old SPF embryonated RIR eggs and titrated in TOCs; the titres of the stock viruses were 4/91(UK) 5.4 log_10_ CD_50_, BeauR-4/91(S) 5.6 log_10_ CD_50_ and M41-CK 6.0 log_10_ CD_50_ in a volume of 1 ml. Five groups (n = 13) of 8-day-old SPF RIR chickens were used for *in vivo* analysis of rIBV BeauR-4/91(S). The chickens were housed in negative-pressure, temperature-controlled HEPA-filtered isolation rooms, with each group housed in a separate room. Three groups of birds were inoculated via the conjunctival (eye drop) and intranasal routes with 3.6 log_10_ CD_50_ of BeauR-4/91(S) in 0.1 ml serum-free BES (N, N-Bis(2-hydroxyethyl)-2-aminoethanesulphonic acid) containing medium. The other two groups were inoculated with serum-free BES medium as controls. Three weeks post-infection, the three groups that had been infected with BeauR-4/91(S) were challenged using 3.6 log_10_ CD_50_ in a total of 0.1 ml with either IBV 4/91(UK), IBV M41-CK or mock-challenged and the two mock-infected groups were either mock challenged or challenged with 4/91(UK); in all cases the challenge viruses were administered via the conjunctival and intranasal routes.

### Assessment of pathogenicity

The clinical signs used to determine pathogenicity were snicking (a sound similar to a sneeze), tracheal rales (a sound emanating from the bronchi, also detected by vibrations when holding a chick), wheezing (dyspoena), nasal discharge, watery eyes and ciliary activity of the trachea [1]. Chicks were observed daily for clinical signs; snicks were independently counted by two persons over a 2 min period. Birds were checked individually for the presence of tracheal rales, nasal discharge, watery eyes and wheezing. Tracheas were removed from three randomly selected chickens from each group at 4, 5 and 6 days post-challenge for assessment of ciliary activity. Ten 1 mm sections were cut from three different regions of each trachea and the level of ciliostasis of each tracheal section was determined using light microscopy.

### Detection of viral RNA from infected chickens

The remaining regions of the tracheas from the infected birds were divided in two, one part was stored in RNA*later*® (Ambion) to be used for RNA extraction, and the other was kept in PBS to be used for virus isolation. For RNA extraction, trachea sections stored in RNA*later*® were placed in RNA lysis buffer followed by disruption and homogenisation in TissueLyser II (Qiagen) for 2 min at 25 Hz. Total RNA was extracted using the RNeasy® kit (Qiagen), following the manufacturer's instructions, and was used as a template for real-time RT-PCR, using primers IBV5′GU391 (_391_GCTTTTGAGCCTAGCGTT_408_) and IBV5′GL533 (_533_GCCATGTTGTCACTGTCTATTG_512_) and a Taqman® dual-labelled probe IBV5′ G probe (FAM-_494_CACCACCAGAACCTGTCACCTC_473_-TAM) [40] using nucleotide positions of IBV M41 (GenBank accession number AY851295). Both primers and probe were synthesised by SIGMA. The 10 µl real-time RT-PCRs consisted of 5 µl of 2x FAST Master mix (Taqman® Fast Universal PCR Master Mix (2x), No AmpErase, Applied Biosystems), 0.25 µl of Multiscribe Enzyme (Applied Biosystems), primers at a final concentration of 1 µM, probe at 5 µM, 2.5 µl of RNA and water. All reactions were performed in triplicate in a 7500FAST Taqman® machine (Applied Biosystems) at 48°C for 30 min; 95°C for 20 sec; 95°C for 3 sec; 40 cycles of 95°C for 3 sec followed by 60°C for 3 sec. Amplification plots were analysed using Applied Biosystems Sequence Detection Software version 1.3.1.21 (2001–2005 Applied Biosystems).

### Viral isolation

Tracheal sections stored in PBS were freeze-thawed and homogenised using the Tissuelyser II (Qiagen). The resulting tracheal suspensions were centrifuged and the supernatants used to infect TOCs. Tracheal suspensions were prepared separately from three birds (except for the mock infected:4/91-challenged group, n = 2) per sampling day (days 4, 5 and 6 after challenge) for the BeauR-4/91(S):4/91 and BeauR-4/91(S):M41 groups. Six TOCs were infected with 100 µl of the corresponding tracheal suspension. After infection at 37°C for 1 h, 0.5 ml of medium was added and the TOCs incubated at 37°C for 7 days during which they were regularly observed for ciliary activity. To compare the ciliary activity results ANOVA analysis was performed followed by the post-hoc Dunnett's multiple comparison test using GraphPad Prism version 5.03 (GraphPad Software Inc. 1992–2010, www.graphpad.com).

## Results

### Generation of a rIBV with the genomic backbone of Beau-R but expressing the S gene from 4/91(UK)

**Figure 2:**
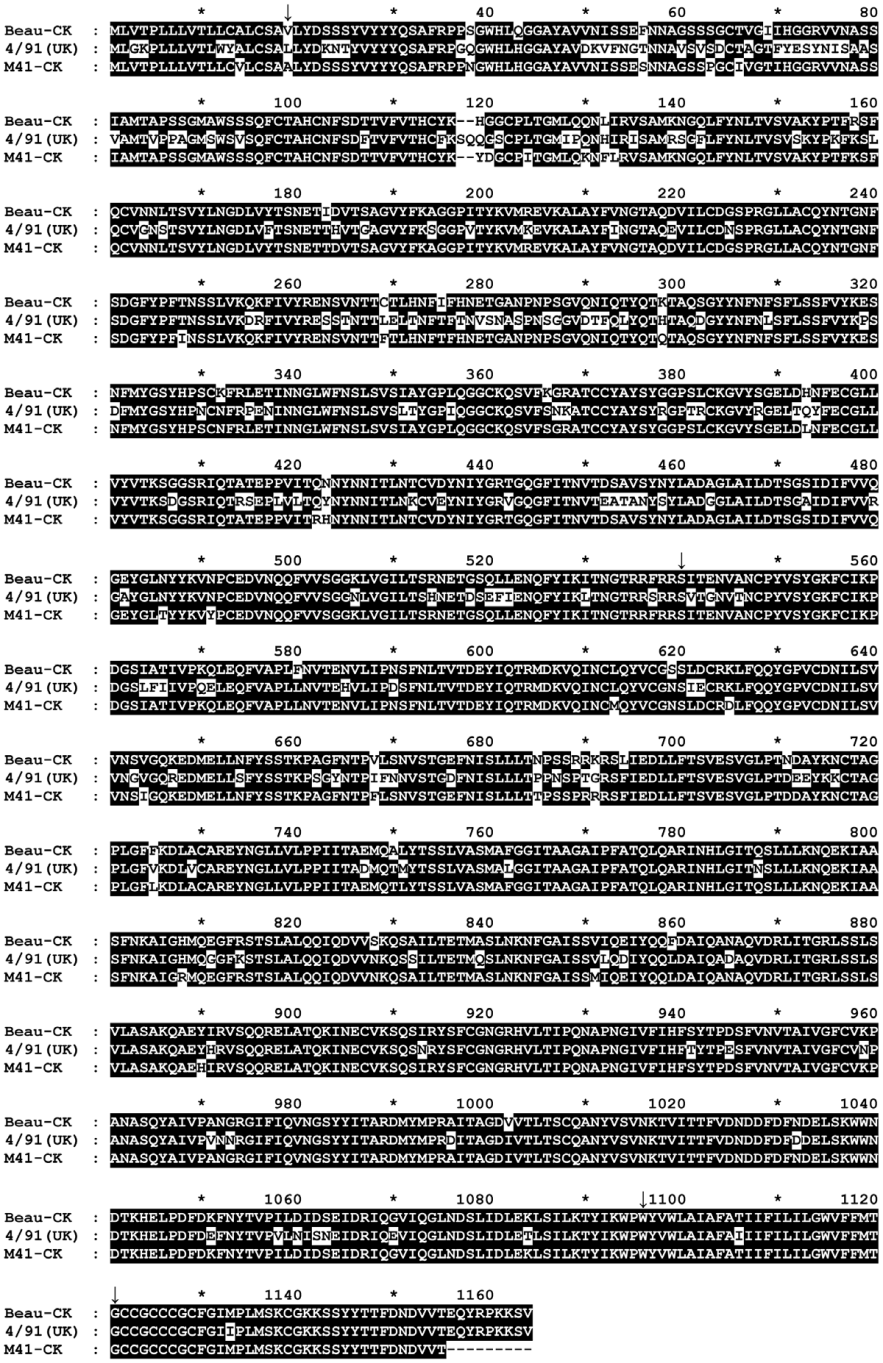
Comparison of the amino acid sequences of the S glycoproteins of IBV strains Beau-R, 4/91(UK) and M41-CK. The sequences were aligned using ClustalX 2.1 [67] and compared using GeneDoc Multiple Sequence Alignment Editor and Shading Utility version 2.7.001 (http://www.nrbsc.org/gfx/genedoc). Amino acids shaded in black represent identical amino acid residues found in each sequence; non-highlighted residues represent differing amino acids. The transition sites between the IBV S glycoprotein domains are marked with an arrow; the signal sequence after position 18, the S1/S2 junction is after position 539, the TM domain starts at position 1096 and the cytoplasmic tail starts at position 1121. The ectodomain is composed of amino acids 19–1095 and the endodomain is composed of amino acids 1096–1164.

Sequence analysis of the IBV 4/91(UK) S gene identified 555 nucleotide differences and a 6-nucleotide insertion when compared to the Beau-R S gene sequence, corresponding to a total of 201 amino acid differences between the two S glycoproteins with two extra amino acids within the 4/91 S glycoprotein (Fig. 2). There is one amino acid difference between the cytoplasmic domains of the two viruses, M^1131^ → I^1133^ for Beau-R and 4/91(UK), respectively. Overall, the primary translation products of the two S genes are 1164 and 1162 amino acids for 4/91(UK) and Beau-R, respectively, with an identity of 82.7% between the two S proteins; 75.3% and 89.1% identity for the S1 and S2 subunits, respectively. A chimaeric S gene, consisting of the signal sequence, ectodomain and transmembrane regions derived from IBV 4/91(UK) and the cytoplasmic tail from Beau-R, was produced. The last 137 nucleotides of the Beaudette S gene, comprising of the cytoplasmic domain, were retained to maintain any interaction [18] of the S protein C-terminal domain with the other Beaudette-derived proteins. The transmembrane regions between the two S glycoproteins only contained one amino acid difference, T^1104^ → I^1106^ for Beau-R and 4/91(UK), respectively. A plasmid, pGPT-4/91S, containing the chimaeric S gene with the above characteristics was generated and used to produce a full-length IBV-derived cDNA, containing the complete genomic sequence of Beau-R but with the chimaeric S gene within the vaccinia virus genome, using the TDS method. This was achieved using a Beau-R-ΔS receiver sequence and a donor sequence in pGPT-4/91S (Fig. 1).

Following TDS, DNA was extracted from four rVVs, potentially containing the BeauR-4/91(S) cDNA. Analysis by PCR, using GPT-specific primers confirmed the loss of the GPT gene following the second TDS recombination event in three of the rVVs. The IBV cDNAs within these rVV DNAs were analysed for the presence of 4/91 S sequence by amplifying a region comprising the S gene. Three rVVs, were then further screened by sequence analysis of different regions of the S1 and S2 subunits of the chimaeric S gene to confirm that an S gene, with the ectodomain sequence from IBV 4/91(UK) had been inserted into the IBV Beaudette genomic sequence lacking an S gene sequence. All three rVVs were found to contain the sequence corresponding to the chimaeric S gene, one rVV, rVV-BeauR-4/91(S), was chosen for further work.

### Recovery of infectious rIBVs from the rVVs

The recovery of an infectious rIBV expressing the chimaeric 4/91 S glycoprotein was attempted using DNA extracted from rVV-BeauR-4/91(S) in CK cells, previously infected with rFPV/T7, to provide T7 RNA polymerase, and co-transfected with the rVV DNA and pCi-Nuc [41] as previously described [27], [28]. The transfected CK cells (P_0_) were incubated for three days and after being filtered to remove any rFPV/T7, the supernatant was used to infect fresh CK cells monolayers. Four days after infection, supernatant and cells were collected (P_1_) and analysed by RT-PCR. After several rescue attempts RT-PCR results showed that there was no rIBV present in P_1_, presumably because IBV 4/91(UK), used as donor of the S gene, is refractory for growth in CK cells. Therefore, 10-day-old embryos were inoculated with cell lysate from the transfected P_0_ CK cells. Allantoic fluid was collected 48 h post infection from the eggs (EP_1_) and total RNA was extracted and analysed by RT-PCR. RT-PCR results were positive for specific 4/91 S products indicative of infectious rIBV. Sequence analysis, using oligonucleotides covering a region from the C-terminus of the replicase gene to the C-terminus of the 3c coding region of gene 3, confirmed that the S gene was from IBV 4/91(UK). The allantoic fluid was then used for further five passages in 10-day-old embryos generating a stock of the rIBV BeauR-4/91(S) that was used for the subsequent experiments.

### Characterisation of rIBV BeauR-4/91(S) for pathogenicity and homologous protection

In order to test the pathogenicity of BeauR-4/91(S) and to determine whether the rIBV is able to induce a protective immune response against challenge with pathogenic 4/91, and to reduce the number of chickens needed, a single experiment was carried out to test these two attributes of the rIBV. The first part of the experiment was to determine whether there were any clinical signs associated with an IBV infection, such as those we knew were associated with IBV 4/91(UK). The second part was to challenge BeauR-4/91(S) vaccinated birds with 4/91(UK) to determine whether the rIBV could induce homologous protection. This part of the experiment required infection of birds with pathogenic 4/91(UK) as a control and was also the control for identifying clinical signs associated with 4/91(UK) for the pathogenicity part of the experiment. In addition, we also challenged chickens that had been vaccinated with BeauR-4/91(S) with a heterologous, different serotype, pathogenic IBV M41-CK. M41 and Beaudette belong to the same serogroup, Massachusetts, and as can be seen from Fig. 2, the S1 part of the M41 S protein sequence differs in a similar way as Beaudette to the 4/91 sequence. Previous studies [42] concluded that the Massachusetts-based vaccines tested did not offer protection against viruses belonging to the 4/91 serogroup.

### Analysis of pathogenicity

**Figure 3:**
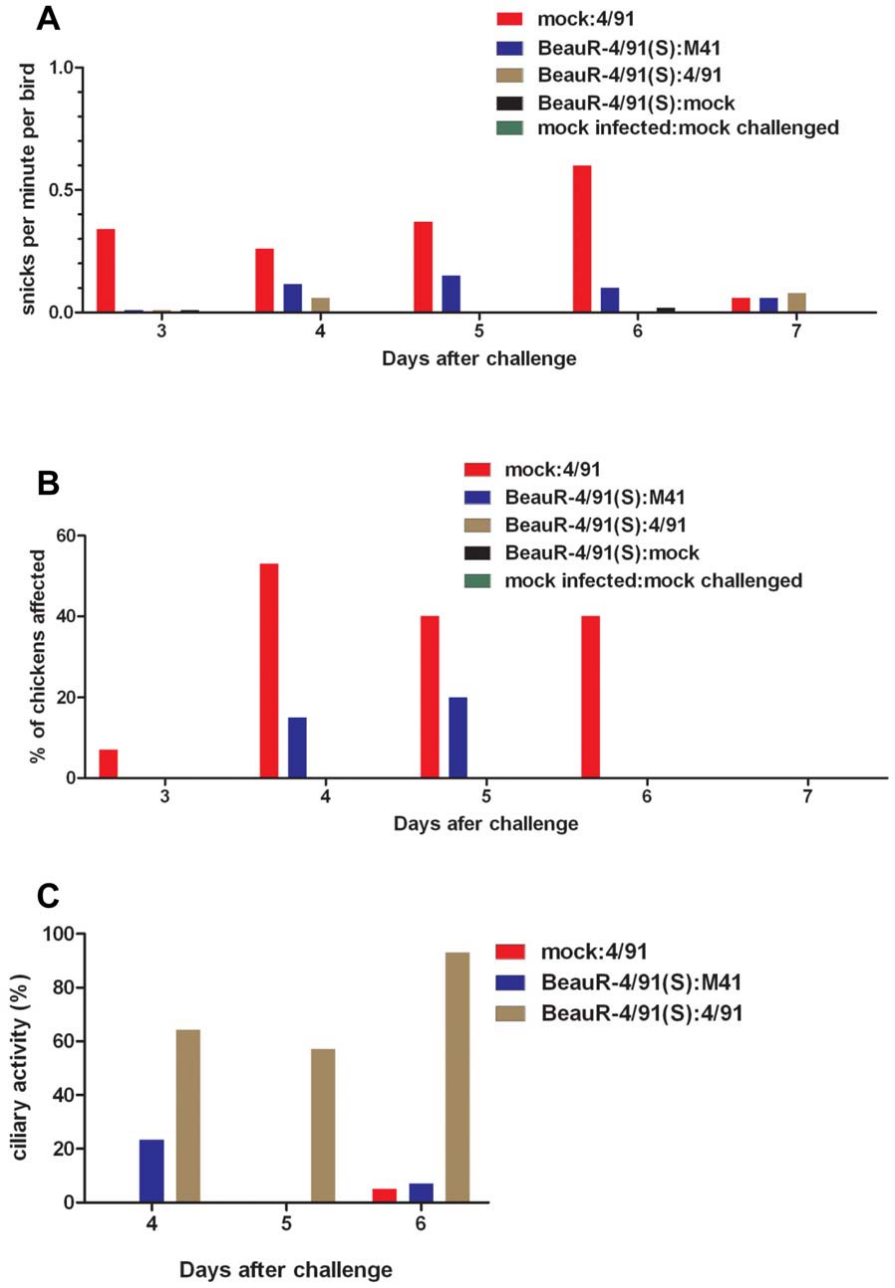
Assessment of clinical signs associated with BeauR-4/91(S)-infected chicks and following challenge with IBV 4/91(UK) or M41-CK. The groups of chickens were assessed for the clinical signs snicking (A) and wheezing (B) following challenge with IBV 4/91(UK) or M41-CK. The levels of these clinical signs were significantly reduced in the BeauR-4/91(S):4/91 and BeauR-4/91(S):M41 groups when compared with the mock:4/91 group (p<0.05). Ciliary activity of the trachea was significantly higher in the BeauR-4/91(S):4/91 group when compared with the control group mock:4/91, indicative of homologous protection (C). Clinical signs were recorded from days 3 to 7 post-challenge. The birds were observed individually except for snicking, where they were observed as a group.

In order to test the pathogenicity of BeauR-4/91(S), three groups of 13 8-day-old SPF chickens were inoculated by eye-drop and intranasally with 3.6 log_10_ CD_50_ of BeauR-4/91(S). Another two groups of 13 birds were infected with 0.1 ml of serum-free medium as controls. The birds were observed for clinical signs, snicking, wheezing and nasal discharge, up to 10 days post-inoculation. Only very low levels of snicking were observed in two of the groups infected with BeauR-4/91(S), 0.07 and 0.03 per min per bird on day 6 and 7 after infection in one group and 0.03 per min per bird on day 6 after infection in the other group, no other clinical signs were observed (data not shown). In contrast, chickens infected with pathogenic 4/91(UK) showed clinical signs associated with IBV from 3 days post infection (Fig. 3A & 3B). These results showed that replacement of the Beau-R S gene with the S gene from 4/91(UK) did not confer pathogenicity to the resulting BeauR-4/91(S) virus.

### Analysis of protection induced by BeauR-4/91(S)

Three weeks after the primary inoculation, the chickens were challenged as follows: of the three groups of chickens previously infected with BeauR-4/91(S), the Beau-4/91(S): 4/91 group was challenged with 3.6 log_10_ CD_50_ of pathogenic IBV 4/91(UK); the Beau-4/91(S):M41 group was challenged with 3.6 log_10_ CD_50_ of pathogenic IBV M41-CK; and the Beau-4/91(S):mock group was mock infected with serum-free medium. Of the two previous control groups, which had not received a primary inoculation, the mock:4/91 group was challenged with 3.6 log_10_ CD_50_ of pathogenic IBV 4/91(UK), and the mock:mock group was challenged with serum-free medium. Observations for clinical signs were carried out daily on each group from three days post-infection. In addition, at 4, 5 and 6 days post-infection the tracheas of three randomly selected chickens from each group were examined for ciliary activity and the presence of IBV.

As anticipated, the mock:4/91 group of chickens that did not receive a primary inoculum with Beau-R-4/91(S) and was subsequently challenged with pathogenic 4/91(UK) showed the highest levels of clinical signs. Snicking rates peaked at 6 days post-challenge (Fig. 3A) with the highest score for wheezing observed at 4 days post-challenge (Fig. 3B). Tracheal rales were observed and one bird showed nasal discharge. Snicking rates differed from 0.6 snicks per min per bird in the mock:4/91 group to 0 snicks on day 6 post-challenge in the BeauR-4/91(S):4/91 group of chickens that were primarily inoculated with Beau-R-4/91(S) and then challenged with pathogenic 4/91(UK) (Fig. 3A) and no wheezing was observed in this group of chickens (Fig. 3B), no other clinical signs, rales or nasal discharge, were observed; indicating that homologous protection had been induced by BeauR-4/91(S). As expected, the control groups, BeauR-4/91(S):mock and mock:mock groups of chickens did not show any clinical signs (Fig. 3A & B).

Consistent with the clinical signs results, the mock:mock group of chickens that was neither primarily inoculated with Beau-R-4/91(S) nor subsequently challenged with a pathogenic IBV showed ciliary activities >95% on day 4 after inoculation (data not shown). The BeauR-4/91(S):mock group of chickens that received the primary inoculation with rIBV BeauR-4/91(S) but was not challenged also showed >95% ciliary activity on day 4 after inoculation (data not shown). In contrast, the mock:4/91 group of chickens that had not been primarily inoculated with Beau-R-4/91(S) but were challenged with pathogenic IBV 4/91(UK) showed the lowest level of ciliary activity, <5% (>95% ciliostasis) on day 4 after challenge with 4/91 (Fig. 3C). The BeauR-4/91(S):4/91 group of chickens that received the primary inoculation with rIBV Beau-R-4/91(S) and subsequently challenged with pathogenic IBV 4/91(UK) showed a high retention of ciliary activity, 88%, by day 6 post-infection when compared to the mock:4/91 group of chickens (Fig. 3C). In other words, the BeauR-4/91(S):4/91 group retained most ciliary activity, indicating that prior infection with BeauR-4/91(S) had induced protection against challenge with pathogenic 4/91(UK).

The BeauR-4/91(S):M41 group of chickens that were primarily inoculated with BeauR-4/91(S) and subsequently challenged with the heterologous serotype of pathogenic IBV M41-CK showed a maximum of 0.15 snicks per bird per min on day 5 post-challenge whereas no snicking was observed in the BeauR-4/91(S):4/91 group of chickens (Fig. 3A). Wheezing was observed in 20% of the BeauR-4/91(S):M41 group of chickens by 5 post-challenge. Previous work showed that M41-CK caused snicking levels peaking at day 5 post-infection (1–2 snicks/min/bird) and wheezing levels of 90%, at day 7 post infection, in infected birds when compared to an avirulent IBV [1], [43]. Therefore, our results suggested that under experimental conditions BeauR-4/91(S) had induced some level of cross protection against M41-CK according to analysis of clinical signs. However, by day 5 post-challenge the BeauR-4/91(S):M41 group of chickens showed 0% ciliary activity (100% ciliostasis), as also observed for the mock:4/91 group of chickens (Fig. 3C), indicating a poor degree of cross protection based on analysis of ciliary activity.

In summary, in terms of clinical observations, snicking and wheezing, birds initially infected with BeauR-4/91(S) and subsequently challenged with 4/91(UK) virus displayed lower levels of clinical signs when compared to birds infected with 4/91(UK) (mock:4/91 group). In contrast, birds inoculated with BeauR-4/91(S) and subsequently challenged with M41-CK were not protected to the same extent. The use of clinical scores and degree of ciliostasis observed, as criteria of pathogenicity, showed there was a strong degree of protection induced by primary inoculation with BeauR-4/91(S) against challenge with homologous pathogenic 4/91(UK) but much less protection when the BeauR-4/91(S) vaccinated chickens were challenged with pathogenic M41-CK belonging to the heterologous serotype. Overall, our results suggest that BeauR-4/91(S) is able to protect chickens against homologous challenge with pathogenic 4/91(UK), but offers less cross protection against challenge with a heterologous serotype such as IBV M41.

### Detection of IBV in the tracheas from the infected chickens

**Figure 4:**
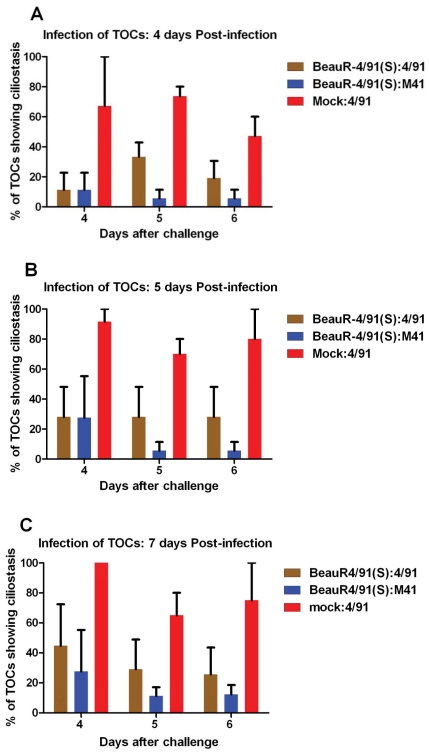
Ciliary activity levels displayed by TOCs infected with trachea-derived suspensions taken from birds infected with BeauR-4/91(S) and challenged with 4/91(UK) or M41-CK compared with non-vaccinated but challenged birds. Ciliary activity levels were significantly higher in BeauR-4/91(S):4/91 and BeauR-4/91(S):M41 groups when compared with mock:4/91 group on each day (p<0.05). Ciliary activity was observed on days 4 (A), 5 (B) and 7 (C) following infection of the TOCs. Each data point represents the average of the readings of TOCs infected with tracheal samples from three birds taken on days 4, 5 and 6 after challenge (average percentage of six TOCs per bird).

In order to determine whether there was any virus in the tracheas of the infected chickens following challenge with pathogenic IBV, tracheal section homogenates derived from infected chickens were used to infect TOCs. Tracheal homogenates derived from tracheas taken from chickens belonging to the mock:4/91, BeauR-4/91(S):4/91 and BeauR-4/91(S):M41 groups at 4, 5 and 6 days after challenge with pathogenic IBV were produced and used to infect TOCs, which were subsequently observed for growth of potential virus as a result of loss of ciliary activity; loss of ciliary activity, >25% ciliostasis, is indicative of IBV being present in the tracheal sample isolated from an infected bird. There was a significant difference (p<0.05) in the ciliary activity displayed by the control birds, mock:4/91 group, compared to the two groups of chickens primarily infected with BeauR-4/91(S) and challenged with either pathogenic IBV 4/91(UK) or M41-CK (Fig. 4). The average levels for ciliostasis observed at days 4, 5 and 7 in the TOCs, after infection with tracheal samples from the challenged birds, were 27±25% for the BeauR-4/91(S):4/91 group and 13±11% for the BeauR-4/91(S):M41 group, whereas an average ciliostasis level of 74±15% was observed for the mock:4/91 group (Fig. 4). This observation suggested that either no challenge virus or very low levels of challenge virus was present in the tracheas of birds initially infected with BeauR-4/91(S) and subsequently challenged with either 4/91(UK) or M41-CK, indicative of the induction of protective immunity by BeauR-4/91(S), including some cross protection.

### Analysis of IBV-RNA present in the tracheas of infected chickens

To check for the presence of 4/91- or M41-derived RNA in the tracheal epithelial cells from the infected chickens following challenge with pathogenic IBV, total RNA was isolated from the epithelial cells and analysed by qRT-PCR. Analysis of the tracheas from the birds belonging to the mock:4/91 group showed they all had IBV-derived RNA present (Table 1). Subsequent sequence analysis confirmed that the IBV RNA present corresponded to pathogenic IBV 4/91(UK). With the exception of one bird, no IBV-derived RNA was detected in the tracheas of birds belonging to the BeauR-4/91(S):4/91 group (Table 1). These results are consistent with the clinical signs observations and with the ciliary activity levels displayed by TOCs infected with the corresponding trachea-derived suspensions prepared from infected chickens and indicate that Beau-R-4/91(S) induced protection against subsequent challenge with pathogenic IBV 4/91(UK). Interestingly, IBV RNA was also only detected in one of the birds belonging to the BeauR-4/91(S):M41 group (Table 1) indicative of some cross protection. Sequence analysis of the RT-PCR product from this sample confirmed that the RNA was derived from M41-CK. This result supports the virus re-isolation results in which infectious IBV was detected in TOCs following infection with tracheal extracts derived from birds sampled from this group.

**Table 1:** Detection of IBV-derived RNA by qRT-PCR in tracheal samples from birds used in the protection study.

Virus Combination	Day 4	Day 5	Day 6
Vaccine BeauR-4/91(S):Challenge M41-CK	**1/3**	**0/3**	**0/3**
Vaccine BeauR-4/91(S):Challenge 4/91(UK)	**1/3**	**0/3**	**0/3**
Mock:Challenge 4/91(UK)	**+**	**+**	**+**

Notes: Tracheas from three birds per group were removed 4, 5 and 6 days after challenge and kept in RNA*later*®. After disruption and homogenisation, RNA was extracted and used as a template for IBV-specific qRT-PCR. Trachea samples were taken from three different birds each day (n = 9), for the control group (mock:4/91) tracheas were taken from one bird per day (n = 3) for assessment. All reactions were performed in triplicate in a 7500FAST Taqman machine. Amplification plots were analysed using Applied Biosystems SDS software. The qRT-PCR method used in this manuscript has previously been assessed for sensitivity, maximum sensitivity being of 100 copies [40].

## Discussion

We have previously constructed a rIBV, BeauR-M41(S), containing the S glycoprotein ectodomain of the pathogenic IBV strain M41-CK within the genomic context of the apathogenic strain, IBV Beau-R, both viruses belong to the same Massachusetts serotype [30]. BeauR-M41(S) was found to acquire the cellular tropism of M41-CK, but remained apathogenic, according to clinical signs, in infected birds [1]. Importantly, birds inoculated with BeauR-M41(S) showed increased levels of protection, when compared to Beau-R, against challenge with M41-CK virus, suggesting that the ectodomain region of the S protein is important in inducing protective immunity in chickens against IBV infection, and therefore represents a region that should be explored in more detail for effective IBV vaccine development. To extend this work, we replaced the ectodomain region of the Beaudette S gene with the sequence of another pathogenic IBV strain, 4/91(UK), which belongs to a different serotype, the 793/B serotype. The first known virus of this IBV serotype was isolated in France in 1985 [44]. In the UK, IBV 4/91 (also known as 793/B and CR88) serotype viruses were first isolated and characterised in 1991 [6], [42], [45] and were subsequently found to be a common pathogen in flocks of chickens in many parts of Western Europe and elsewhere by the late 1990s [46]. Despite the availability of an attenuated vaccine against 4/91 serotype viruses, recent studies suggest that viruses belonging to the 4/91 serogroup are still prevalent in European flocks [47]–[50] and that their continued presence is still an important concern for the poultry industries in many different countries [6].

The rIBV BeauR-4/91(S) grew in eggs and in TOCs, but was not able to produce infectious virus in CK cells suggesting that it acquired the tropism characteristics of the IBV donor strain 4/91(UK), which unlike Beau-R, is refractory for replication in CK cells, providing further evidence that the S protein is the major determinant of tropism and host range. These results support those from our previous work in which the ectodomain of the S gene in Beaudette was replaced with that from an IBV strain of the same serotype, M41-CK. The recombinant IBV, BeauR-M41(S), was shown to have its *in vitro* host range restricted to those cells in which M41-CK could grow [30].

Similar to the observations with BeauR-M41(S), our results showed that BeauR-4/91(S) was also apathogenic, supporting our previous observation that an S gene from a pathogenic strain, even from a different serotype, is not sufficient to confer pathogenicity. Consistent with these findings, pathogenicity studies done with a chimaeric IBV in which the replicase gene was from the apathogenic IBV Beaudette, but the rest of the genome was from the pathogenic M41-CK strain, showed that this chimaeric virus was still not pathogenic, indicating that determinants of pathogenicity reside within the replicase gene [43].

Work by others has shown that IBV H120, a major Massachusetts serotype vaccine, induced poor protection against challenge with a 4/91 virus [42]. The amino acid sequences of the S proteins of Beau-R and 4/91(UK) differ by 25% and 11% for the S1 and S2 subunits, respectively. Since it is the S1 subunit that induces virus neutralising antibodies and protective immune responses [51]–[54], this suggests that the ability to induce protective responses against 4/91 may reside in some of the 25% amino acid differences between this strain and Beau-R. Similar to our results for BeauR-M41(S), our protection studies in this work showed that BeauR-4/91(S), which contains the 4/91(UK) S protein ectodomain (comprising S1 and most of S2), conferred protection against challenge with 4/91(UK). The criteria used to measure pathogenicity were observation of clinical signs (snicking, wheezing), ciliary activity in the trachea, presence of virus-derived RNA (by qRT-PCR) in tracheas and virus isolation from tracheal samples. According to clinical signs observations, prior inoculation of chickens with BeauR-4/91(S) resulted in protection against challenge with 4/91(UK). Analysis of the tracheas from the BeauR-4/91(S):4/91 challenge group of chickens showed a significant retention of ciliary activity levels when compared to the very low levels of ciliary activity observed in tracheal samples from the mock:4/91 group. This observation was supported by qRT-PCR analysis on RNA extracted from trachea samples. Only one chicken was positive for the detection of viral RNA in the trachea samples from chickens in the BeauR-4/91(S):4/91 challenge group. Furthermore, there was clearly a reduction in the levels of ciliostasis displayed by TOCs infected with potential virus present in tracheal suspensions prepared from the BeauR-4/91(S):4/91 group when compared to the mock:4/91 group. Interestingly, comparison of the data indicated there was more ciliostasis observed from tracheal samples from the BeauR-4/91(S):M41 group than the BeauR-4/91(S):4/91 group, however, the overall level of ciliostasis observed from either group is outside the level expected for presence of virus. The degree of cross protection among different IBV serotypes is generally low and decreases as the amino acid differences between the S protein increases [55], [56]. By replacing the S gene of the apathogenic Beau-R, from the Massachusetts serotype, with that from 4/91(UK), from the 793/B serotype, we have produced an apathogenic virus that induces protection against challenge with a pathogenic virus from a different serotype, 4/91(UK). Demonstrating and confirming that serotype is associated exclusively with the S glycoprotein irrespective of the genotype of the IBV strain.

Although challenge of BeauR-4/91(S) vaccinated birds with M41-CK resulted in ciliostasis, observation of clinical signs, qRT-PCR and virus isolation suggested there was a low level of protection against challenge with the heterologous M41-CK, even though 4/91(UK) and M41-CK are from different serotypes. In our previous work, Hodgson *et al.* [1], we similarly showed that no virus was recovered from the tracheas of chickens inoculated with Beau-R and challenged with M41-CK, even though the level of ciliostasis was high (>80%). Most IBV serotypes differ by 20% to 25% from each other in the S1 sequence at amino acid level [57]–[62], some differing only by 2% [63]. The S1 subunit of M41-CK and 4/91(UK) differ by 25.5% at the amino acid level. Although cross protection amongst different serotypes is generally poor, some cross protection between different serotypes has been observed previously. An IBV 4/91 vaccine was shown to protect against challenge (as judged by retention of ciliary activity) with heterologous serotypes Arkansas, D207 and the Brazilian isolates 50/96, 57/96, 62/96 and 64/96. However, no protection was observed against challenge with M41, as judged by loss of ciliary activity, clinical signs and virus isolation wee not assessed [64]. In addition, the IBV vaccine H120 conferred some protection against challenge viruses, from the Belgium B1648, French 84084 and French 84221 serotypes of IBV [64]. Some heterologous protection against M41 was observed in birds vaccinated with the heterologous serotype UK/6/82, despite the 20% difference in S1 [55]. Vaccination with TM-86w, classified as a JP-II genotype on the basis of the sequence of the N-terminus of the spike protein, conferred protection against challenge with 4/91, despite being classified as being a different genotype, as indicated by respiratory symptoms, ciliostasis and virus re-isolation from tracheal swabs [65].

Although we did not include a mock:M41 control group, the levels of snicking and wheezing observed in the BeauR-4/91(S):M41 group were less than would have been expected from a mock:M41 group as observed from previous experiments [1], [43], indicating some degree of cross protection, which was supported by the results of virus re-isolation and analysis of viral RNA by qRT-PCR. Interestingly, although vaccination with BeauR-4/91(S) did induce a degree of cross protection against challenge with M41-CK, as judged by reduced clinical signs, no virus isolation and no detection of viral RNA, there was extensive loss of ciliary activity. We also observed this phenomenon in our previous work when birds vaccinated with Beau-R had been challenged with M41-CK [1]. Work by Marquardt *et al*. [66] showed that virus was isolated from tracheas exhibiting ciliostasis following a heterologous challenge. However, up to three passages in TOCs was required to reveal the presence of virus, indicating that the amount of virus present in the tracheas had been very low. We can only speculate why heterologous vaccination sometimes results in protection, as judged by reduced clinical signs and absence of detectable virus in the trachea whilst at the same time appearing not to have induced protection against heterologous challenge, when based on loss of ciliary activity (ciliostasis). Retention of tracheal ciliary activity has long been used to assess cross protection [55], [64]. Our finding and those of Marquardt *et al*. [66] do not argue against the use of this criterion as a correlate of protection. The importance of an intact respiratory epithelium would be manifest more in the field than under laboratory conditions. Partial cross protection, based on clinical signs and virus recovery, observed in experiments performed under laboratory conditions in experimental animal facilities would be expected to be sometimes insufficient to protect against economic losses in the field. The damage to the respiratory epithelium, as reflected by observation of ciliostasis, would enable pathogenic strains of bacteria e.g. *E.coli*, when present, to cause additional pathology.

Our previous work used a rIBV expressing a heterologous S glycoprotein from IBV M41-CK that belongs to the same serogroup as the recipient virus, Beau-R, which differs by only 5% with respect to the amino acid sequence. We have now extended this work by demonstrating that expression of an heterologous S glycoprotein from a different serogroup, which differs by 25% in the amino acid sequence to either Beau-R or M41-CK S glycoproteins, conferred homologous protection against challenge with pathogenic 4/91(UK). In addition, according to clinical signs, detection of virus RNA and re-isolation of virus there was a degree of heterologous protection, under experimental conditions, against challenge with M41-CK, an IBV isolate from a different serotype.

## References

[1] Hodgson T, Casais R, Dove B, Britton P, Cavanagh D (2004). Recombinant infectious bronchitis coronavirus Beaudette with the spike protein gene of the pathogenic M41 strain remains attenuated but induces protective immunity.. J Virol.

[2] Carstens EB (2010). Ratification vote on taxonomic proposals to the International Committee on Taxonomy of Viruses (2009).. Arch Virol.

[3] Cavanagh D (2005). Coronaviruses in poultry and other birds.. Avian Pathol.

[4] Cavanagh D, Gelb J, Saif YM (2008). Infectious Bronchitis.. Diseases of Poultry. 12th ed.

[5] Jones RC (2010). Viral respiratory diseases (ILT, aMPV infections, IB): are they ever under control?. Br Poult Sci.

[6] de Wit JJ, Cook JKA, van der Heijden HMJF (2011). Infectious bronchitis virus variants: a review of the history, current situation and control measures.. Avian Pathol.

[7] Cavanagh D, Naqi S, Saif YM, Barnes HJ, Glisson JR, Fadly AM, McDougald LR (2003). Infectious bronchitis.. Diseases of Poultry. 11 ed.

[8] Britton P, Cavanagh D, Thiel V (2007). Avian coronavirus diseases and infectious bronchitis vaccine development.. Coronaviruses: Molecular and Cellular Biology.

[9] Cook JKA, Mockett APA, Siddell SG (1995). Epidemiology of infectious bronchitis virus.. The Coronaviridae.

[10] Cavanagh D (2007). Coronavirus avian infectious bronchitis virus.. Vet Res.

[11] Lambrechts C, Pensaert M, Ducatelle R (1993). Challenge experiments to evaluate cross-protection induced at the trachea and kidney level by vaccine strains and Belgian nephropathogenic isolates of avian infectious bronchitis virus.. Avian Pathol.

[12] Ziegler AF, Ladman BS, Dunn PA, Schneider A, Davison S (2002). Nephropathogenic infectious bronchitis in Pennsylvania chickens 1997–2000.. Avian Dis.

[13] Britton P, Cavanagh D, Perlman S, Gallagher T, Snijder EJ (2008). Nidovirus genome organization and expression mechanisms.. Nidoviruses.

[14] de Vries AAF, Horzinek MC, Rottier PJM, de Groot RJ (1997). The genome organisation of the Nidovirales: Similarities and differences between Arteri-, Toro- and Coronaviruses.. Semin Virol.

[15] Lai MM, Cavanagh D (1997). The molecular biology of coronaviruses.. Adv Virus Res.

[16] Vennema H, Rottier PJ, Heijnen L, Godeke GJ, Horzinek MC (1990). Biosynthesis and function of the coronavirus spike protein.. Adv Exp Med Biol.

[17] Delmas B, Laude H (1990). Assembly of coronavirus spike protein into trimers and its role in epitope expression.. J Virol.

[18] Godeke GJ, de Haan CA, Rossen JW, Vennema H, Rottier PJ (2000). Assembly of spikes into coronavirus particles is mediated by the carboxy-terminal domain of the spike protein.. J Virol.

[19] Gallagher TM, Buchmeier MJ (2001). Coronavirus spike proteins in viral entry and pathogenesis.. Virology.

[20] Koch G, Hartog L, Kant A, van Roozelaar DJ (1990). Antigenic domains of the peplomer protein of avian infectious bronchitis virus: correlation with biological function.. J Gen Virol.

[21] Schultze B, Cavanagh D, Herrler G (1992). Neuraminidase treatment of avian infectious bronchitis coronavirus reveals a hemagglutinating activity that is dependent on sialic acid-containing receptors on erythrocytes.. Virology.

[22] Luo ZL, Weiss SR (1998). Roles in cell-to-cell fusion of two conserved hydrophobic regions in the murine coronavirus spike protein.. Virology.

[23] de Groot RJ, Lujtjes W, Horzinek MC, van der Zeijst BAM, Spaan WJ (1987). Evidence for a coiled-coil structure in the spike proteins of coronaviruses.. J Mol Biol.

[24] Tripet B, Howard MW, Jobling M, Holmes RK, Holmes KV (2004). Structural characterization of the SARS-coronavirus spike S fusion protein core.. J Biol Chem.

[25] Guo Y, Tisoncik J, McReynolds S, Farzan M, Prabhakar BS (2009). Identification of a new region of SARS-CoV S protein critical for viral entry.. J Mol Biol.

[26] Shulla A, Gallagher T (2009). Role of spike protein endodomains in regulating coronavirus entry.. J Biol Chem.

[27] Casais R, Thiel V, Siddell SG, Cavanagh D, Britton P (2001). Reverse genetics system for the avian coronavirus infectious bronchitis virus.. J Virol.

[28] Britton P, Evans S, Dove B, Davies M, Casais R (2005). Generation of a recombinant avian coronavirus infectious bronchitis virus using transient dominant selection.. J Virol Meth.

[29] Armesto M, Casais R, Cavanagh D, Britton P, Cavanagh D (2008). Transient dominant selection for the modification and generation of recombinant infectious bronchitis coronaviruses.. SARS- and Other Coronaviruses: Laboratory Protocols: Humana Press.

[30] Casais R, Dove B, Cavanagh D, Britton P (2003). Recombinant avian infectious bronchitis virus expressing a heterologous spike gene demonstrates that the spike protein is a determinant of cell tropism.. J Virol.

[31] Beaudette FR, Hudson CB (1937). Cultivation of the virus of infectious bronchitis.. J Am Vet Med Assoc.

[32] Bijlenga G, Cook JKA, Gelb J, de Wit JJ (2004). Development and use of the H strain of avian infectious bronchitis virus from The Netherlands as a vaccine: a review.. Avian Pathol.

[33] Davelaar FG, Kouwenhoven B (1976). Changes in the Harderian gland of the chicken following conjunctival and intranasal infection with infectious bronchitis virus in one- and 20-day old chickens.. Avian Pathol.

[34] Davelaar FG, Kouwenhoven B (1980). Effect of the removal of the Harderian gland in 1-day-old chicks immunity following IB vaccination.. Avian Pathol.

[35] Toro H, Godoy V, Larenas J, Reyes E, Kaleta EF (1996). Avian infectious bronchitis: viral persistence in the harderian gland and histological changes after eyedrop vaccination.. Avian Dis.

[36] Cook JKA, Darbyshire JH, Peters RW (1976). The use of chicken tracheal organ cultures for the isolation and assay of avian infectious bronchitis virus.. Arch Virol.

[37] Darbyshire JH, Rowell JG, Cook JKA, Peters RW (1979). Taxonomic studies on strains of avian infectious bronchitis virus using neutralisation tests in tracheal organ cultures.. Arch Virol.

[38] Jones BV, Hennion RM (2008). The preparation of chicken tracheal organ cultures for virus isolation, propagation, and titration.. Meth Mol Biol.

[39] Bonfield JK, Smith KF, Staden R (1995). A new DNA sequence assembly program.. Nucl Acids Res.

[40] Callison SA, Hilt DA, Boynton TO, Sample BF, Robison R (2006). Development and evaluation of a real-time Taqman RT-PCR assay for the detection of infectious bronchitis virus from infected chickens.. J Virol Meth.

[41] Hiscox JA, Wurm T, Wilson L, Britton P, Cavanagh D (2001). The coronavirus infectious bronchitis virus nucleoprotein localizes to the nucleolus.. J Virol.

[42] Parsons D, Ellis MM, Cavanagh D, Cook JKA (1992). Characterisation of an avian infectious bronchitis virus isolated from IB-vaccinated broiler breeder flocks.. Vet Rec.

[43] Armesto M, Cavanagh D, Britton P (2009). The replicase gene of avian coronavirus infectious bronchitis virus is a determinant of pathogenicity.. PLoS ONE.

[44] Picault JP, Drouin P, Lamande J, Allee C, Toux JY (1995). L'epizootie recente de bronchite infectieuse aviaire en France: importance, evolution et etiologie.. Proceedings of the 1eres Journée de la Recherche Avicole, Angers 28–29 March.

[45] Gough RE, Randall CJ, Dagless M, Alexander DJ, Cox WJ (1992). A ‘new’ strain of infectious bronchitis virus infecting domestic fowl in Great Britain.. Vet Rec.

[46] Cook JK, Orbell SJ, Woods MA, Huggins MB (1996). A survey of the presence of a new infectious bronchitis virus designated 4/91 (793B).. Vet Rec.

[47] Worthington KJ, Currie RJ, Jones RC (2008). A reverse transcriptase-polymerase chain reaction survey of infectious bronchitis virus genotypes in Western Europe from 2002 to 2006.. Avian Pathol.

[48] Dolz R, Pujols J, Ordóñez G, Porta R, Majó N (2008). Molecular epidemiology and evolution of avian infectious bronchitis virus in Spain over a fourteen-year period.. Virology.

[49] Bochkov YA, Batchenko GV, Shcherbakova LO, Borisov AV, Drygin VV (2006). Molecular epizootiology of avian infectious bronchitis in Russia.. Avian Pathol.

[50] Bochkov YA, Tosi G, Massi P, Drygin VV (2007). Phylogenetic analysis of partial S1 and N gene sequences of infectious bronchitis virus isolates from Italy revealed genetic diversity and recombination.. Virus Genes.

[51] Cavanagh D, Davis PJ (1986). Coronavirus IBV: removal of spike glycopolypeptide S1 by urea abolishes infectivity and haemagglutination but not attachment to cells.. J Gen Virol.

[52] Ignjatovic J, Galli L (1994). The S1 glycoprotein but not the N or M proteins of avian infectious bronchitis virus induces protection in vaccinated chickens.. Arch Virol.

[53] Johnson MA, Pooley C, Ignjatovic J, Tyack SG (2003). A recombinant fowl adenovirus expressing the S1 gene of infectious bronchitis virus protects against challenge with infectious bronchitis virus.. Vaccine.

[54] Song CS, Lee YJ, Lee CW, Sung HW, Kim JH (1998). Induction of protective immunity in chickens vaccinated with infectious bronchitis virus S1 glycoprotein expressed by a recombinant baculovirus.. J Gen Virol.

[55] Cavanagh D, Ellis MM, Cook JKA (1997). Relationship between sequence variation in the S1 spike protein of infectious bronchitis virus and the extent of cross-protection *in vivo*.. Avian Pathol.

[56] Cavanagh D (2003). Severe acute respiratory syndrome vaccine development: experiences of vaccination against avian infectious bronchitis coronavirus.. Avian Pathol.

[57] Adzhar A, Gough RE, Haydon D, Shaw K, Britton P (1997). Molecular analysis of the 793/B serotype of infectious bronchitis virus in Great Britain.. Avian Pathol.

[58] Farsang A, Ros C, Renström LHM, Baule C, Soós T (2002). Molecular epizootiology of infectious bronchitis virus in Sweden indicating the involvement of a vaccine strain.. Avian Pathol.

[59] Keeler CL, Reed KL, Nix WA, Gelb J (1998). Serotype identification of avian infectious bronchitis virus by RT-PCR of the peplomer (S-1) gene.. Avian Dis.

[60] Kingham BF, Keeler CL, Nix WA, Ladman BS, Gelb J (2000). Identification of avian infectious bronchitis virus by direct automated cycle sequencing of the S-1 gene.. Avian Dis.

[61] Lee CW, Hilt DA, Jackwood MW (2001). Identification and analysis of the Georgia 98 serotype, a new serotype of infectious bronchitis virus.. Avian Dis.

[62] Sapats SI, Ashton F, Wright PJ, Ignjatovic J (1996). Sequence analysis of the S1 glycoprotein of infectious bronchitis viruses: Identification of a novel genotypic group in Australia.. J Gen Virol.

[63] Cavanagh D, Davis PJ, Cook JKA, Li D, Kant A (1992). Location of the amino-acid differences in the S1 spike glycoprotein subunit of closely related serotypes of infectious-bronchitis virus.. Avian Pathol.

[64] Cook JKA, Orbell SJ, Woods MA, Huggins MB (1999). Breadth of protection of the respiratory tract provided by different live-attenuated infectious bronchitis vaccines against challenge with infectious bronchitis viruses of heterologous serotypes.. Avian Pathol.

[65] Shimazaki Y, Horiuchi T, Harada M, Tanimura C, Seki Y (2008). Isolation of 4/91 type of infectious bronchitis virus as a new variant in Japan and efficacy of vaccination against 4/91 type field isolate.. Avian Dis.

[66] Marquardt WW, Kadavil SK, Snyder DB (1982). Comparison of ciliary activity and virus recovery from tracheas of chickens and humoral immunity after inoculation with serotypes of avian infectious bronchitis virus.. Avian Dis.

[67] Larkin MA, Blackshields G, Brown NP, Chenna R, McGettigan PA (2007). Clustal W and Clustal X version 2.0.. Bioinformatics.

